# Comparison of Disease Phenotype and Course among Elderly- and Early-Onset Inflammatory Bowel Diseases in the Middle East

**DOI:** 10.34172/aim.2023.73

**Published:** 2023-09-01

**Authors:** Hasan Vosoghinia, Bahar Saberzadeh-Ardestani, Amir Anushiravani, Fariborz Mansour-Ghanaei, Hafez Fakheri, Homayoon Vahedi, Farshad Sheikhesmaeili, Abbas Yazdanbod, Seyed Hamid Moosavy, Iradj Maleki, Siavosh Nasseri-Moghaddam, Bardia Khosravi, Masoud Malekzadeh, Amir Kasaeian, Sudabeh Alatab, Anahita Sadeghi, Shadi Kolahdoozan, Mohammad Amani, Seyedeh Naeimeh Saberhosseini, Maryam Rayatpisheh, Mitra Ahadi, Jean-Frederic Colombel, Ryan C. Ungaro, Ali Reza Sima, Reza Malekzadeh

**Affiliations:** ^1^Gastroenterology and Hepatology Department, Faculty of Medicine, Ghaem Hospital, Mashhad, Iran; ^2^Digestive Disease Research Center, Digestive Disease Research Institute, Tehran University of Medical Sciences, Tehran, Iran; ^3^Gastrointestinal and Liver Diseases Research Center, Guilan University of Medical Sciences, Rasht, Iran; ^4^Gut and Liver Research Center, Mazandaran University of Medical Sciences, Sari, Iran; ^5^Liver and Digestive Research Center, Kurdistan University of Medical Sciences, Sanandaj, Iran; ^6^Gastroenterology and Hepatology Department, Digestive Diseases Research Center, Ardabil University of Medical Sciences, Ardabil, Iran; ^7^Shahid Mohammadi Hospital, Hormozgan University of Medical Sciences, Bandar Abbas, Iran; ^8^Hematology, Oncology and Stem Cell Transplantation Research Center, Tehran University of Medical Sciences, Tehran, Iran; ^9^Mashhad University of Medical Sciences, Mashhad, Iran; ^10^The Henry D. Janowitz Division of Gastroenterology Icahn School of Medicine at Mount Sinai, New York, USA; ^11^Sasan Alborz Biomedical Research Center, Masoud Gastroenterology and Hepatology Center, Tehran, Iran

**Keywords:** Crohn’s disease, Early-onset, Elderly-onset, Inflammatory bowel disease, Ulcerative colitis

## Abstract

**Background:**

It is unknown if the clinical manifestations and phenotype of disease are comparable between early- and elderly-onset inflammatory bowel disease (IBD). We aimed to seek differences in disease phenotype, course, complications, and treatment between early- and elderly-onset IBD patients.

**Methods:**

This retrospective cohort study on registered IBD patients in the Iranian Registry of Crohn’s and Colitis (IRCC) compared demographics, disease phenotype, disease activity, IBD-related surgery and medications between early- and elderly-onset IBD. A generalized linear regression model was used to investigate the relative risk of age at diagnosis adjusted for gender and disease duration for the outcomes.

**Results:**

From 10048 IBD patients, 749 with early-onset (7.5%), and 472 (4.7%) elderly-onset IBD were enrolled: 855 (63.1%) ulcerative colitis (UC) and 366 (26.9%) Crohn’s disease (CD). Left-sided colitis was more frequent among elderly-onset UC patients (*P*<0.001). Ileum and ileocolonic locations were the most common types in elderly-onset and early-onset CD patients, respectively. In comparison with elderly-onset UC, early-onset cases more often used prednisolone (22.1% vs. 11.4%, *P*=0.001), immunomodulators (44.9% vs 25.2%, *P*<0.001) and anti-tumor necrosis factors (TNF) (20.1% vs 11.9%, *P*=0.002). Elderly-onset UC patients had 0.7 times lower risk of aggressive phenotype (95%CI:0.6‒0.9, *P*=0.005). Early-onset CD was associated with higher use of prednisolone (27.7% vs 8.1%, *P*<0.001), immunomodulators (58.7% vs 41.8%, *P*=0.005) and anti-TNF (49.6% vs 35.4%, *P*=0.006).

**Conclusion:**

Early-onset IBD was associated with a more aggressive phenotype and higher prednisolone, immunomodulators, and anti-TNF use.

## Introduction

 A combination of genetic background, abnormal immune reaction to the gut microbiota, and environmental risk factors lead to inflammatory bowel disease (IBD), including ulcerative colitis (UC) and Crohn’s disease (CD).^1^ Middle Eastern and East European countries have reported a progressive rise in the incidence of IBD.^2^ Moreover, the prevalence of IBD has been increasing over the past decades in most regions and total years lived with disability attributed to IBD have shown a rising trend.^2^

 IBD is usually diagnosed in the third and fourth decades of life, with a smaller second peak in the 1960s and 1970s.^3^ Previous studies have reported between 7‒30% of IBD cases as elderly-onset.^4-9^ The reason for the variety in the age of onset is not clearly understood. One hypothesis has proposed that genetic factors may play a more decisive role in early-onset IBD, while environmental factors may be more critical in elderly-onset IBD.^10^ There is also an ongoing debate where elderly-onset IBD is considered as a separate condition.^11^

 Clinical manifestations and phenotypes of IBD may differ based on the age of onset.^12^ Elderly-onset CD usually presents with inflammatory behavior, while the early-onset CD has shown a penetrating behavior with more frequent small bowel involvement.^13^ Moreover, patients with early-onset IBD have shown more disease extension during their disease course.^14^ Several studies have suggested that early-onset IBD patients need treatment with biologic agents and immunomodulators due to a more aggressive disease.^15^

 Many uncertainties exist regarding the differences in clinical manifestations and disease course, phenotype, and treatment among elderly- and early-onset IBD patients. There has been an increasing incidence of early-onset IBD in the last few decades.^16,17^ Moreover, with the aging population, elderly-onset IBD has become a major concern that will grow with increasing human life expectancy.^18^ Due to the typical exclusion of elderly patients from clinical trials, understanding the difference between these groups is essential for appropriate disease management.^19^ We aimed to seek differences in disease course, phenotype, treatment, and complications among elderly- and early-onset IBD patients.

## Materials and Methods

 This retrospective cohort study used data of IBD patients who were registered in the Iranian Registry of Crohn’s and Colitis (IRCC) from December 2017 until March 2022 supported by the Iranian Crohn’s and Colitis Foundation.^20^ The diagnosis of IBD was confirmed with clinical, endoscopic, radiological, and pathological characteristics based on the World Gastroenterology guidelines in 2015.^21^

 Gastroenterologists completed a questionnaire according to the patient’s clinical records about the age of diagnosis, type of IBD, history of IBD-related surgery, extent of UC (according to the Montreal classification system as the maximum extent at any time point, classified as proctitis, left colitis and pancolitis), Crohn’s disease location (either ileal, ileocolonic, colonic and others), behavior (either non-stricturing, non-penetrating, fistulizing, and stricturing), extraintestinal manifestations (either autoimmune hepatitis [AIH], ankylosing spondylitis [AS], sclerosing cholangitis [PSC], uveitis, peripheral arthritis, erythema nodosum, and pyoderma gangrenosum [PG]).

 Then, a research assistant interviewed patients by telephone to gather additional information about sex, education, ethnicity, medications prescribed at any time since the disease diagnosis (e.g., 5-aminosalicylic acid [5-ASA], anti-tumor necrosis factor [anti-TNF], immunomodulators, including azathioprine/mercaptopurine/methotrexate, and prednisolone). Disease activity during the previous six months was assessed with the Manitoba IBD Index (MIBDI) using a 6-level patient-reported response (scores less than four were considered as active disease). Patients’ disease activity in the previous two weeks was recorded by a patient-reported outcome measure (PROM) IBD-control questionnaire (score 14 or less considered as an active disease).^22,23^ We defined aggressive phenotype as either active disease in the last six months, history of surgery for IBD, or anti-TNF usage.

 We defined early-onset IBD as at 18 years of age or under, while elderly-onset was considered at 60 years of age or above.

###  Statistical Analysis

 We presented categorical variables as numbers and percentages and compared the distribution of categorical variables between early- and elderly-onset IBD cases using Pearson χ2 and Fisher exact test (if needed). We used this univariable analysis to investigate the association between age at diagnosis and outcomes. Then, we selected variables with *P* value < 0.1, and we entered them into the generalized linear model to investigate the relative risk of age at diagnosis adjusted for gender and disease duration for the outcomes. For the generalized linear model, dummy variables were generated for non-binary outcomes. We conducted all statistical analyses using the Stata 11.2 edition (StataCorp. 2011, Stata Statistical Software, Release 12, College Station, TX) for Windows. *P* values < 0.05 were considered significant.

## Results

 Overall, 10 048 IBD patients were recruited in the Iranian Registry of Crohn’s and Colitis (IRCC) from December 2017 until March 2022. Of these patients, 1221 patients were enrolled in this study, including 749 patients (7.5%) with age at diagnosis below 18 years and 472 (4.7%) cases who were diagnosed above 60 years of age. Among them, 855 (63.1%) had UC and 366 (26.9%) had CD.

###  Early- and elderly-onset UC

Table 1 summarizes the demographic features of the 855 UC patients registered in this research. The mean age was 42.7 years (SD, 24.5), and 469 (54.9%) were male. The average disease duration was 10.1 (SD, 9.5), and 4.8 (SD, 4.3) years in early-onset (n = 515), and elderly-onset (n = 340) UC patients (*P* < 0.001).

**Table 1:** Clinical Characteristics and Demographic Features of Ulcerative Colitis Patients Stratified by Age at Diagnosis

**Variables**^a,b^	**N=855**	**Ulcerative colitis**		***P *** **Value**
		**Early-onset (n=515)**	**Elderly-onset (n=340)**	
Age, *n* (%)				
0‒9	21 (2.5)	21 (4.1)	0 (0.0)	< 0.001
10‒19	133 (15.8)	133 (25.9)	0 (0.0)	
20‒29	240 (28.8)	240 (46.6)	0 (0.0)	
30‒39	89 (10.6)	89 (17.3)	0 (0.0)	
40‒49	22 (2.6)	22 (4.3)	0 (0.0)	
50‒59	7 (0.8)	7 (1.4)	0 (0.0)	
60‒69	144 (17.2)	2 (0.4)	142 (43.9)	
70‒79	126 (15.1)	0 (0.0)	126 (39.1)	
80‒89	52 (6.2)	0 (0.0)	52 (16.1)	
> 90	3 (0.4)	0 (0.0)	3 (0.9)	
Sex, *n* (%)				
Female	386 (45.2)	245 (47.6)	141 (41.5)	0.079
Male	469 (54.9)	270 (52.4)	199 (58.5)	
Years of disease duration, mean (SD)	8.1 (8.3)	10.1 (9.5)	4.8 (4.3)	< 0.001
Education, *n* (%)				
Illiterate	91 (10.6)	10 (1.9)	81 (23.8)	0.001
1-12 years	484 (56.6)	295 (57.3)	189 (55.6)	
> 12 years	280 (32.8)	210 (40.8)	70 (20.6)	
Ethnicity, *n* (%)				
Persian	471 (55.1)	275 (53.4)	196 (57.6)	0.001
Azeri	168 (19.6)	118 (22.9)	50 (14.7)	
Lor	23 (2.7)	18 (3.5)	5 (1.5)	
Kurd	86 (10.1)	54 (10.5)	32 (9.4)	
Arab	3 (0.4)	1 (0.2)	2 (0.6)	
Turkmen	2 (0.2)	2 (0.4)	0 (0.0)	
Other	102 (11.9)	47 (9.1)	55 (16.2)	

^a^ Percentages do not include missing values and were calculated for each row by dividing on the corresponding N value. ^b^ Percentages from each subcategory may not add up to the exact number of total reported cases due to missing values and/or non-mutually exclusive variables.

 The association between age at diagnosis and outcomes in UC patients is shown in Table 2. While extraintestinal manifestations were seen in 46.4% of early-onset UC patients, its prevalence was 38.2% in elderly-onset cases (*P* = 0.018). UC extent was significantly different between early- and elderly-onset UC cases, with more left-sided colitis among elderly-onset cases (*P*< 0.001). While 22.1% of early-onset UC patients were currently using or had taken prednisolone, this rate was 11.4% in elderly-onset UC patients (*P* = 0.001). Immunomodulators were used more commonly in the early-onset UC patients compared with the elderly-onset patients (44.9% vs. 25.2%, *P* < 0.001). Also, anti-TNFs use was more prevalent among the early-onset UC patients in comparison with the elderly-onset UC cases (20.1% vs. 11.9%, *P* = 0.002).

**Table 2:** Association of Ulcerative Colitis Clinical Phenotype, Disease Course, and Outcomes with Patients’ Age at Diagnosis (Early- and Elderly-Onset)

**Variables**^a,b^	**N=855**	**Ulcerative Colitis**		***P *** **Value**
		**Early-Onset (n=515)**	**Elderly-Onset (n=340)**	
Extraintestinal Manifestations, n (%)	369 (43.2)	239 (46.4)	130 (38.2)	0.018
Extent, *n* (%)				
Proctitis	65 (14.9)	33 (13.4)	32 (17.1)	< 0.001
Left sided colitis	157 (36.2)	69 (27.9)	88 (47.1)	
Pancolitis	212 (48.9)	145 (58.7)	67 (35.8)	
IBD medication, *n* (%)				
Prednisolone	100 (17.4)	71 (22.1)	29 (11.4)	0.001
5-ASA	779 (93.6)	477 (93.9)	302 (93.2)	0.692
Immunomodulator	274 (37.1)	200 (44.9)	74 (25.2)	< 0.001
Anti-TNF	141 (16.9)	102 (20.1)	39 (11.9)	0.002
Active disease during the past 2 weeks, *n* (%)	40 (4.7)	27 (5.2)	13 (3.8)	0.336
Active disease during 6 months, *n* (%)	147 (17.2)	91 (17.7)	56 (16.5)	0.649
IBD-related surgeries, *n* (%)	27 (3.2)	18 (3.5)	9 (2.7)	0.488
Aggressive phenotype, *n* (%)	268 (31.4)	175 (33.9)	93 (27.4)	0.041

IBD, Inflammatory Bowel Disease; 5-ASA, 5-Aminosalicylic Acid; Anti-TNF, Anti-Tumor necrosis factor. ^a^ Percentages do not include missing values and were calculated for each row by dividing on the corresponding N value. ^b^ Percentages from each subcategory may not add up to the exact number of total reported cases due to missing values and/or non-mutually exclusive variable.

 Early-onset UC patients did not differ in disease activity (either in the previous two weeks or past 6 months) and IBD-related surgery compared with elderly-onset UC patients. The aggressive phenotype was more prevalent in early-onset UC patients in comparison with elderly-onset UC patients (33.9% vs. 27.4%, *P* = 0.041).

**Figure 1:**
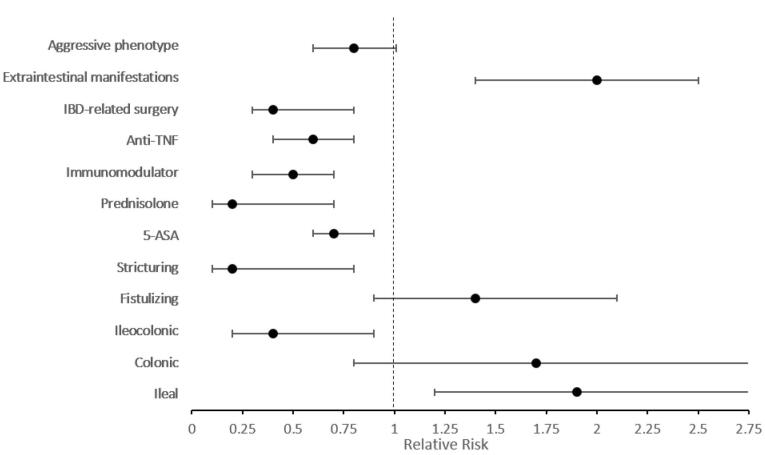
Relative Risk of Outcomes in Patients with Elderly-Onset Ulcerative Colitis in Comparison with Early-Onset Patients Adjusted by Disease Duration and Gender

Figure 1 shows the relative risk of outcomes after adjusting for disease duration and gender. The risk of left colitis was 1.7 times higher among elderly-onset UC patients (95% CI: 1.3‒2.3, *P* < 0.001). Regarding medication, prednisolone, immunomodulator, and anti-TNF use remained significantly lower among elderly-onset cases. Moreover, elderly-onset UC patients had 0.7 times lower risk of aggressive phenotype in comparison with early-onset patients (95% CI: 0.6‒0.9, *P* = 0.005) (Supplementary file 1, Table S1).

###  Early- and Elderly-onset CD

 The demographic features of the 366 CD patients are displayed in Table 3. The average age was 36.5 years (SD, 20.2), and 214 (58.5%) were male. Early-onset CD patients’ (n = 234) mean disease duration was 10.1 (SD, 9.1) years, and it was 3.3 (SD, 2.9) years in elderly-onset cases (n = 132) (*P* < 0.001).

**Table 3:** Clinical Characteristics and Demographic Features of Crohn's Disease Patients Stratified by Age at Diagnosis

**Variables**^a,b^	**N=366**	**Crohn's Disease**		***P *** **Value**
		**Early-Onset (n=234)**	**Elderly-Onset (n=132)**	
Age, *n* (%)				
0‒10	3 (0.9)	3 (1.3)	0 (0.0)	< 0.001
11‒20	66 (20.8)	66 (28.2)	0 (0.0)	
21‒30	106 (33.5)	106 (45.3)	0 (0.0)	
31‒40	52 (16.4)	52 (22.2)	0 (0.0)	
41‒50	6 (1.9)	6 (2.6)	0 (0.0)	
51‒60	1 (0.3)	1 (0.4)	0 (0.0)	
61‒70	46 (14.5)	0 (0.0)	46 (55.4)	
71‒80	30 (9.5)	0 (0.0)	30 (36.2)	
81‒90	7 (2.2)	0 (0.0)	7 (8.4)	
> 90	0 (0.0)	0 (0.0)	0 (0.0)	
Sex, *n* (%)				
Female	152 (41.5)	96 (41.0)	56 (42.4)	0.794
Male	214 (58.5)	138 (59.0)	76 (57.6)	
Years of disease duration, mean (SD)	8.4 (8.5)	10.1 (9.1)	3.3 (2.9)	< 0.001
Education, *n* (%)				
Illiterate	15 (4.1)	1 (0.4)	14 (10.6)	< 0.001
1-12 years	204 (55.7)	137 (58.6)	67 (50.8)	
> 12 years	147 (40.2)	96 (41.0)	51 (38.6)	
Ethnicity, *n* (%)				
Persian	220 (60.1)	139 (59.4)	81 (61.4)	0.923
Azeri	67 (18.3)	43 (18.4)	24 (18.2)	
Lor	19 (5.2)	13 (5.6)	6 (4.6)	
Kurd	18 (4.9)	10 (4.3)	8 (6.1)	
Arab	3 (0.8)	2 (0.9)	1 (0.8)	
Turkmen	4 (1.1)	2 (0.9)	2 (1.5)	
Other	35 (9.6)	25 (10.7)	10 (7.6)	

^a^Percentages do not include missing values and were calculated for each row by dividing on the corresponding N value. ^b^Percentages from each subcategory may not add up to the exact number of total reported cases due to missing values and/or non-mutually exclusive variables.

Table 4 presents the association between age at diagnosis and outcomes in patients with CD. Elderly-onset CD cases had a higher prevalence of extraintestinal manifestations (62.2% vs. 49.6% in early-onset). The location and behavior of CD were significantly different between the two groups (*P* = 0.002 and *P* < 0.001, respectively). While ileum involvement was the most common type in elderly-onset CD patients, ileocolonic involvement was the most prevalent among early-onset patients.

**Table 4:** Association of Crohn's Disease Clinical Phenotype, Disease Course and Outcomes with Patients' Age at Diagnosis (Early- and Elderly-Onset).

**Variables**^a,b^	**N=366**	**Crohn's Disease**		***P *** **Value**
		**Early-Onset (n=234)**	**Elderly-Onset (n=132)**	
Extraintestinal manifestations, *n* (%)	198 (54.1)	116 (49.6)	82 (62.2)	0.021
Location, *n* (%)				
Ileal	57 (39.3)	32 (32.3)	25 (54.4)	0.002
Colonic	27 (18.6)	16 (16.2)	11 (23.9)	
Ileocolonic	60 (41.4)	51 (51.5)	9 (19.6)	
Upper GI	1 (0.7)	0 (0.0)	1 (2.2)	
Disease behavior, *n* (%)				
Fistulizing	72 (56.3)	45 (50.0)	27 (71.1)	< 0.001
Stricturing	42 (32.8)	39 (43.3)	3 (7.9)	
IBD medication, *n* (%)				
Prednisolone	43 (19.9)	36 (27.7)	7 (8.1)	< 0.001
5-ASA	283 (80.2)	192 (83.8)	91 (73.4)	0.019
Immunomodulator	161 (52.6)	115 (58.7)	46 (41.8)	0.005
Anti-TNF	162 (45.2)	117 (49.6)	45 (35.4)	0.006
Active disease during the past 2 weeks, n (%)	31 (8.5)	22 (9.4)	9 (6.8)	0.394
Active disease during 6 months, *n* (%)	89 (24.3)	54 (23.1)	35 (26.5)	0.462
IBD-related surgeries, n (%)	51 (13.9)	40 (17.1)	11 (8.3)	0.020
Aggressive phenotype, *n* (%)	218 (59.6)	148 (63.3)	70 (53.0)	0.056

IBD, inflammatory bowel disease; 5-ASA, 5-aminosalicylic acid; Anti-TNF, anti-tumor necrosis factor. ^a^ Percentages do not include missing values and were calculated for each row by dividing on the corresponding N value. ^b^Percentages from each subcategory may not add up to the exact number of total reported cases due to missing values and/or non-mutually exclusive variables.

 Prednisolone use was seen in 27.7% of early-onset CD patients; however, this rate was 8.1% in elderly-onset CD patients (*P* < 0.001). Immunomodulators were used more commonly for early-onset CD patients than the elderly-onset cases (58.7% vs. 41.8%, *P* = 0.005). Also, anti-TNF use was more prevalent among the early-onset CD patients than the elderly-onset CD cases (49.6% vs. 35.4%, *P* = 0.006).

 Early- and elderly-onset CD patients had similar disease activity during the previous two weeks and six months. IBD-related surgery was more prevalent in early-onset CD patients in comparison with elderly-onset CD patients (17.1% vs 8.3%, *P* = 0.020).

**Figure 2:**
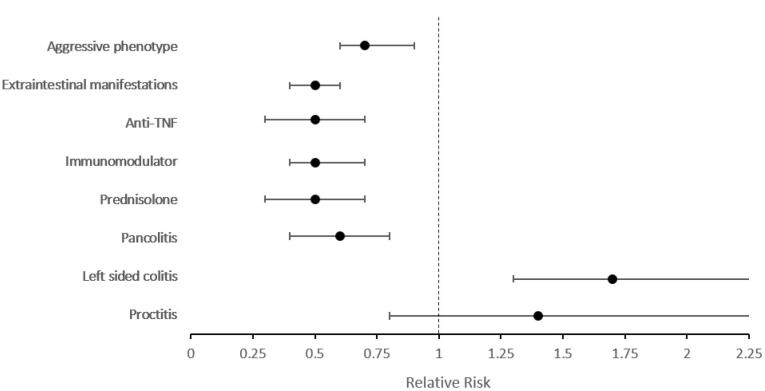
Relative Risk of Outcomes in Patients with Elderly-Onset Crohn’s Disease in Comparison with Early-Onset Patients Adjusted by Disease Duration and Gender

Figure 2 demonstrates the relative risk of outcomes when adjusted for disease duration and gender. Involvement of the ileum was 1.9 times more frequent in elderly-onset CD patients (95% CI: 1.2‒3.3, *P* = 0.009), while the elderly-onset CD cases had a much lower risk (0.2) of stricture formation compared with early cases (95% CI: 0.1‒0.8, *P* = 0.018). Regarding treatment, prednisolone, immunomodulator, and anti-TNF usage remained significantly lower among elderly-onset patients. However, the aggressive phenotype was similar between early- and elderly-onset CD cases (*P* = 0.069) (Supplementary file 1, Table S2).

## Discussion

 This retrospective cohort study shows the course, clinical phenotype, treatment, and complications in elderly-onset IBD compared with early-onset cases adjusted for disease duration and gender. Left-sided colitis was more common in elderly-onset UC patients, and early-onset UC cases had more aggressive disease. Ileal Crohn’s disease was more prevalent among elderly-onset cases, and early-onset cases showed a stricturing phenotype more frequently. The use of prednisolone, immunomodulators, and anti-TNF was more common among early-onset IBD patients.

 The prevalence of elderly-onset IBD has been reported from 7% to 30% in different countries.^4-9,19^ While 30% of patients in South Korea^24^ and 10%‒30% of cases in Europe^5,7,25,26^ were elderly-onset, this rate was 7%‒13% in China^8,9^ and Canada,^27,28^ 10‒15% in US^4,6^ and 17% in Australia.^29^ In our study, out of 10 048 IBD patients in the IRCC cohort, 472 (4.7%) had elderly-onset IBD. The prevalence of elderly-onset IBD was lower in Iran in comparison to other countries.

###  Early- and Elderly-onset UC

 Understanding the clinical phenotype and disease course of IBD in elderly-onset cases is critical for reducing misdiagnosis and delayed diagnosis.^30^ In this study, extraintestinal manifestations were less common in elderly-onset UC cases; this is in line with previous reports.^5,31,32^ Similar to previous studies, we found that left colitis was more common in elderly-onset UC cases. Early-onset patients had more extensive disease.^5,19,31-33^ The aggressive phenotype was more prevalent in early-onset UC patients. This finding is similar to previous studies.^33,34^

 There is controversy regarding the association of age at diagnosis and IBD-related surgery in the literature. While some studies reported higher IBD-related surgery rates among elderly-onset UC patients,^19,30-32^ others reported the same or even lower rates.^5,18,35^ It has been hypothesized that more severe initial presentation that has been reported in some studies could lead to a higher surgery rate among elderly-onset patients.^30^ Moreover, the physician’s attempt to exclude malignancy could also play a role in the higher rate of decision for surgery.^30^ However, in our study, there was no difference between IBD-related surgery in elderly- and early-onset UC patients. This disparity could be associated with the genetic and environmental differences across populations.

 Drug metabolism and clearance are affected by aging, and there has been concern about the complications of immunosuppressive therapy in the elderly.^30,36^ In this study, prednisolone, immunomodulator, and anti-TNF use was significantly lower among elderly-onset patients; this is similar to previous studies.^5,18,19,31-33^ This observation could be considered from two perspectives. The physicians’ hesitation in prescribing immunosuppressive therapy for the elderly could play a role in this finding. However, since disease activity was controlled in the elderly-onset cases, this finding could be attributed to this population’s less severe disease course.

###  Early- and Elderly-onset CD

 We observed higher rates of inflammatory behavior in elderly-onset CD and stricturing behavior in early-onset CD patients; this is similar to some studies,^19,30,37^ but in contrast with others.^5,32^ Early-onset CD patients showed more ileocolonic involvement, while elderly-onset CD patients had ileal involvement more commonly. This finding is in contrast with previous studies which report higher colonic involvement in elderly-onset CD cases^5,19,30,31,37^ Moreover, extraintestinal manifestations were more prevalent among elderly-onset CD patients. This finding is in contrast with previous studies.^5,31,32^ These differences could be due to the genetic variations across populations.

 Prednisolone, immunomodulators, and anti-TNF use was reported to be less prevalent among the elderly-onset CD cases.^5,37^ This is similar to our result. On the other hand, Kedia et al did not report a significant difference in treatment with oral corticosteroids and anti-TNF therapies between elderly-onset CD patients and other CD cases.^19^ The difference in these drugs among the elderly-onset group could be attributed to either a less severe disease course or higher rate of drug-related complications.^36^ Disease activity during the previous six months and two weeks were similar between early- and elderly-onset CD patients. Previous studies have reported similar results.^18,19,30,35,37^ Further studies are needed to investigate drug-related complications in detail.

 In this study, we included cases were from all provinces of Iran. Moreover, this is the most extensive study in the Middle East to compare elderly- and early-onset IBD clinical phenotype and disease courses. The retrospective cohort study design makes it possible to explore causality and relative risk of outcomes based on age at diagnosis. Moreover, sex and disease duration were adjusted between the two groups to reduce the confounders’ effect. Also, we defined outcomes following international standardized guidelines and used valid questionnaires.

 Finally, some limitations need to be considered. Although this was a national study, there was discrepancy in participation rates across provinces, and most of the centers were referral centers which could lead to selection bias. Also, this study might suffer from recall bias since the research assistant asked retrospective questions from the participants by measuring patient-reported disease activity. Furthermore, we defined treatment by the history of medication use, and the duration and dosage of medications were not reviewed in this study.

## Conclusion

 In conclusion, the prevalence of elderly-onset IBD was 4.7% in the IRCC cohort. Extraintestinal manifestations were seen more commonly in the early-onset UC patients. Left-sided colitis was more common in elderly-onset UC patients. Ilium involvement was more prevalent among elderly-onset CD cases and early-onset cases showed a stricturing phenotype more frequently. The use of prednisolone, immunomodulators, and anti-TNF was more common among early-onset IBD patients. Early-onset IBD had more severe outcomes. While IBD-related surgery was similar between the early- and elderly-onset UC patients, higher rates of IBD-related surgery was observed among early-onset CD cases. We suggest a more thorough study on the types of surgery and therapy-related complications in the future.

## Supplementary Files

Supplementary file 1 contains Tables S1 and S2.
