# Supplementary material for: Comparison of Disease Phenotype and Course among Elderly- and Early-Onset Inflammatory Bowel Diseases in the Middle East
Source: Arch Iran Med. 2023 Sep 1;26(9):481–8. doi: 10.34172/aim.2023.73 (PMC10862057; doi:10.34172/aim.2023.73)
Supplement: Supplementary file 1 — contains Tables S1 and S2. [file aim-26-481-s001.pdf]

Arch Iran Med. September 2023;26(9):481-S1.

10.34172/aim.2023.73

Supplementary file 1

Table S1. Relative risk of outcomes in patients with Ulcerative colitis based on patients' age at diagnosis (early- and elderly-onset) adjusted by disease duration and gender.

Variable (Referent group)	Proctitis		Left-sided Colitis		Pancolitis	
	Relative Risk (95% CI)	*P*-Value	Relative Risk (95% CI)	*P*-Value	Relative Risk (95% CI)	*P*-Value
Age at diagnosis (early-onset)	1.4 (0.8, 2.3)	0.685	1.7 (1.3, 2.3)	<0.001*	0.6 (0.4, 0.8)	<0.001*
Years of disease duration	0.9 (0.9, 1.01)	0.254	0.9 (0.9, 0.9)	<0.001*	0.9 (0.9, 0.9)	<0.001*
Gender (Female)	1.3 (0.8, 2.1)	0.267	0.9 (0.7, 1.3)	0.749	1.2 (0.9, 1.5)	0.142
**Variable (Referent group)**	**Prednisolone**		**Immunomodulator**		**Anti-TNF**	
	**Relative Risk (95% CI)**	***P*-Value**	**Relative Risk (95% CI)**	***P*-Value**	**Relative Risk (95% CI)**	***P*-Value**
Age at diagnosis (early-onset)	0.5 (0.3, 0.7)	0.001*	0.5 (0.4, 0.7)	<0.001*	0.5 (0.3, 0.7)	<0.001*
Years of disease duration	1.0 (0.9,1.0)	0.462	0.9 (0.9, 1.1)	0.937	1.0 (0.9, 1.0)	0.162
Gender (Female)	0.8 (0.6, 1.2)	0.457	0.9 (0.7,1.1)	0.502	0.9 (0.7, 1.1)	0.502
**Variable (Referent group)**	**Extraintestinal Manifestations**		**Aggressive Phenotype**			
	**Relative Risk (95% CI)**	***P*-Value**	**Relative Risk (95% CI)**	***P*-Value**		
Age at diagnosis (early-onset)	0.5 (0.4, 0.6)	<0.001*	0.7 (0.6, 0.9)	0.005*		
Years of disease duration	1.01 (1.01, 1.02)	<0.001*	0.9 (0.9, 1.0)	0.141		
Gender (Female)	0.97 (0.97, 0.97)	<0.001*	1.1 (0.9, 1.1)	0.341		

5-ASA; 5-Aminosalicylic Acid; Anti-TNF; Anti-Tumor necrosis factor

Table S2. Relative risk of outcomes in patients with Crohn's disease based on patients' age at diagnosis (early- and elderly-onset) adjusted by disease duration and gender.

Variable (Referent group)	Ileal		Colonic		Ileocolonic		IBD-related Surgery	
	**Relative Risk (95% CI)**	***P*-Value**	**Relative Risk (95% CI)**	***P*-Value**	**Relative Risk (95% CI)**	***P*-Value**	**Relative Risk (95% CI)**	***P*-Value**
Age at diagnosis (early-onset)	1.9 (1.2, 3.3)	0.009*	1.7 (0.8, 3.7)	0.190	0.4 (0.2, 0.9)	0.018*	0.4 (0.3, 0.8)	0.003*
Years of disease duration	0.9 (0.9, 1.0)	0.419	0.9 (0.9, 1.0)	0.301	0.9 (0.9, 1.0)	0.126	1.03 (1.03, 1.03)	<0.001*
Gender (Female)	1.1 (0.6, 1.8)	0.749	0.7 (0.3, 1.4)	0.318	1.2 (0.7, 1.8)	0.530	0.7 (0.7, 0.7)	<0.001*
**Variable (Referent group)**	**Fistulizing**		**Stricturing**		**Extraintestinal Manifestations**		**Aggressive phenotype**	
	**Relative Risk (95% CI)**	***P*-Value**	**Relative Risk (95% CI)**	***P*-Value**	**Relative Risk (95% CI)**	***P*-Value**	**Relative Risk (95% CI)**	***P*-Value**
Age at diagnosis (early-onset)	1.4 (0.9, 2.1)	0.168	0.2 (0.1, 0.8)	0.018*	2.0 (1.4, 2.5)	<0.001*	0.8 (0.6, 1.01)	0.069
Years of disease duration	0.9 (0.9, 0.9)	<0.020*	1.0 (0.9, 1.0)	0.550	1.01 (1.01, 1.01)	<0.001*	1.0 (0.9, 1.0)	0.509
Gender (Female)	0.9 (0.6, 1.5)	0.881	0.6 (0.3, 1.1)	0.064	1.03 (1.03, 1.03)	<0.001*	1.1 (0.9, 1,3)	0.259
**Variable (Referent group)**	**5-ASA**		**Prednisolone**		**Immunomodulator**		**Anti-TNF**	
	**Relative Risk (95% CI)**	***P*-Value**	**Relative Risk (95% CI)**	***P*-Value**	**Relative Risk (95% CI)**	***P*-Value**	**Relative Risk (95% CI)**	***P*-Value**
Age at diagnosis (early-onset)	0.7 (0.6, 0.9)	<0.001*	0.2 (0.1, 0.7)	0.005*	0.5 (0.3, 0.7)	0.001*	0.6 (0.4, 0.8)	0.005*
Years of disease duration	1.003 (1.003, 1.003)	<0.001*	1.01 (0.9, 1.03)	0.356	1.01 (1.0, 1.02)	0.043*	1.0 (0.9, 1.0)	0.267
Gender (Female)	1.02 (1.02, 1.02)	<0.001*	0.9 (0.5, 1.5)	0.706	1.2 (0.9, 1.5)	0.118	1.1 (0.8, 1.4)	0.417

5-ASA, 5-Aminosalicylic Acid; Anti-TNF, Anti-Tumor necrosis factor
